# Identification and management of alcohol use and illicit substance use in outpatient psychiatric clinics in Sweden: a national survey of clinic directors and staff

**DOI:** 10.1186/s13722-019-0140-x

**Published:** 2019-03-06

**Authors:** Christopher Sundström, Elisabeth Petersén, Kristina Sinadinovic, Peter Gustafsson, Anne H. Berman

**Affiliations:** 1 Department of Clinical Neuroscience, Center for Psychiatry Research, Karolinska Institutet and Stockholm Health Care Services, Stockholm County Council, Norra Stationsgatan 69, 113 64 Stockholm, Sweden; 2 Stockholm Center for Dependency Disorders, 118 95 Stockholm, Sweden; 3 Department of Sociology, Stockholm University, 114 18 Stockholm, Sweden

**Keywords:** Substance use, Alcohol use, Psychiatry, Guideline adherence, Screening, Brief intervention

## Abstract

**Background:**

Swedish national guidelines recommend that all health care settings systematically screen patients for alcohol use and illicit substance use. When hazardous use is identified, it should immediately be addressed, preferably through brief interventions (BI). It is well known that the prevalence of alcohol use and illicit substance use among psychiatric patients is high, but it is not known to what extent screening and BI are routinely carried out in such clinics.

**Methods:**

Two online surveys investigating the use of screening and BI for alcohol and illicit substances were constructed; one for psychiatric outpatient clinic directors and one for staff at these clinics. The main analyses were calculated as simple frequencies. In secondary analyses, we investigated the associations between substance abuse training, type of clinic and screening/BI delivery. For these analyses, the Chi square test was used.

**Results:**

Most clinic directors reported that they have guidelines to screen for alcohol (93.1%) and illicit substance use (78.9%) at initial assessment. Fifty percent reported having guidelines for delivering BI when identifying hazardous alcohol use (35.9% for hazardous illicit substance use). Among staff, 66.6% reported always screening for alcohol use and 57.8% reported always screening for illicit substance use at initial assessment. Further, 36.7% reported that they usually deliver BI when identifying hazardous alcohol use (35.7% for hazardous illicit substance use). Secondary analyses indicated that staff with substance abuse training were significantly more likely to screen for alcohol use than staff without such training. Further, staff at psychosis clinics were significantly less likely to screen for both alcohol and substance use than staff at both general and specialist psychiatric clinics.

**Conclusions:**

Most clinic directors reported having clear guidelines for staff to screen for alcohol use and illicit substance use, but fewer staff members than expected indicated that these guidelines were adhered to. Providing training about substance use disorders for staff may increase use of screening for alcohol use, and psychosis clinics may need to improve their screening routines.

**Electronic supplementary material:**

The online version of this article (10.1186/s13722-019-0140-x) contains supplementary material, which is available to authorized users.

## Background

It is well known from epidemiological studies that people with a psychiatric disorder frequently have a concurrent substance abuse or dependence concerning either alcohol or illicit substances [1–3]. Prevalence of substance abuse or dependence among persons in the general population with anxiety or depression is estimated to be 25–30%, with markedly higher levels among those with more severe psychiatric disorders such as schizophrenia [1]. The prevalence is believed to be just as high among patients in psychiatric clinics [4–7]. Individuals with concurrent psychiatric disorder and substance use problems have a worse treatment prognosis [8] and an increased risk of later relapse in their psychiatric disorder [9]. *Hazardous alcohol use*, a drinking pattern not deemed to be a fully developed alcohol abuse or dependence but with potential to lead to adverse consequences, is also problematic in a psychiatric setting. Excessive drinking commonly interferes with psychosocial functioning and raises the risk of subsequent escalation of alcohol problems. In fact, even moderate alcohol intake has a negative impact on clinical course and response to treatment [10, 11], and may interact negatively with common psychiatric medications such as fluoxetine [11] and benzodiazepines [12]. Reduced hazardous drinking among psychiatric patients has been associated with more rapid symptom improvement in anxiety and depression [13]. *Hazardous illicit substance use* is not an equally established concept, but it is not controversial to suggest that sporadic use of illicit substances also has negative implications for treatment and recovery from psychiatric disorders.

The importance of detecting both hazardous alcohol use and abuse or dependence among patients in health care settings outside specialized addiction care was established by the World Health Organization with the development of the Alcohol Use Disorders Identification Test (AUDIT), a questionnaire developed specifically to facilitate identification of alcohol problems within primary care [14]. This opportunistic approach to identifying hazardous alcohol use as well as abuse and dependence has subsequently been incorporated into a host of national guidelines such as those issued by the National Institute for Health and Excellence (NICE) in the UK [15], the National Institute on Alcohol Abuse and Alcoholism (NIAAA) in the United States [16] and the National Board of Health and Welfare in Sweden [17]. These guidelines propose that health care staff should routinely carry out alcohol screening, preferably with a validated questionnaire such as the AUDIT. When alcohol abuse or dependence is identified, the patient is to be offered referral to appropriate treatment, and when hazardous use of alcohol is identified, this should be addressed immediately within the current health service with structured feedback, preferably by means of a brief intervention (BI). BI is a single-session treatment aimed at helping hazardous drinkers moderate their drinking by providing screening, feedback and brief advice regarding their alcohol consumption. BI has been shown to have small but stable effects in the primary care setting [18] and in the emergency department setting [19]. Although research on BI in the psychiatric setting is scarce, a few randomized trials have demonstrated that, although efficacy in terms of effects on alcohol consumption is not yet clear, BI is at least feasible and safe to use in this population [20–23]. In Sweden, some progress has been made in evaluating the implementation of national guidelines for substance abuse and dependence care through quality indicators from the National Patient Registry and the National Quality Registry for Dependency (SBR), but so far no systematic information on screening and BI within psychiatry is available [24]. The importance of detecting illicit substance use has not been placed on the wider healthcare agenda to the same degree as alcohol, but the Drug Use Disorders Identification Test (DUDIT) [25, 26], a parallel instrument to the AUDIT, is nationally recommended by the National Board of Health and Welfare in Sweden for identifying illicit substance use in a variety of health care settings as well as within specialized addiction care [27]. In the UK, the 2011 NICE guidelines on coexisting severe mental illness (psychosis) and substance misuse recommend assessment and management of illicit substance use in wider healthcare settings, specifically stipulating that individuals seeking help within psychiatry should not be excluded due to illicit substance use [28]. In the US, the current focus lies more on ensuring a continuum of care for individuals with illicit substance use [29], with limited research published on identifying and managing illicit substance use within psychiatry [13]. Regarding the efficacy of BI for illicit substance use, the evidence is unclear. Few studies have been conducted on BI for illicit substance use, and a minority of these have been conducted in clinical settings. Two large randomized trials have been conducted in a primary care population: one of these identified a reduced drug use effect following BI [30], while another did not [31]. Recent pooled evidence suggests that interventions delivered via the internet could yield small, but significant overall effects beyond control conditions including BI [32]. To our knowledge, no studies have investigated BI for illicit substance use in the context of psychiatry.

Given the high levels of comorbidity and negative implications of both hazardous use and substance abuse/dependence for treatment outcomes, it would seem essential for any psychiatric clinic to have an effective strategy in place to detect and assess these problems among patients. This could be achieved by routinely screening all patients for alcohol and illicit substance use at initial assessment. Such a strategy could identify many patients early on, facilitating collaboration or referral to appropriate substance use treatment. Even better, it could also identify patients with these problems early enough to allow clinicians to offer on-site treatment such as BI or other suitable measures to address and reduce substance use. However, we have found no existing research on the extent to which screening for alcohol and illicit substance use is conducted in psychiatric outpatient clinics, nor to what extent patients with hazardous use of alcohol or illicit substances are offered BI.

The purpose of this study was to investigate current national practices for identifying and managing hazardous substance use and diagnosed substance abuse/dependence (both alcohol and illicit substances) among patients in psychiatric outpatient clinics in Sweden. Specific research questions were:

1. To what extent do psychiatric outpatient clinics in Sweden have specific guidelines for staff to a) screen for alcohol use and illicit substance use during initial assessment and b) use brief intervention when appropriate?
2. To what extent do staff in psychiatric outpatient clinics in Sweden a) screen for alcohol use and illicit substance use during initial assessment and b) use brief intervention when appropriate?

## Methods

### Design, participants and setting

The study consisted of two separate cross-sectional online surveys, one for directors at outpatient psychiatric clinics and the second for staff with patient contact at these clinics. Ethical review resulted in a consultative statement by the Regional Ethics Review Board in Stockholm, Sweden, stating that no ethical review was deemed necessary (2012/1695-31/5).

### Procedure

The psychiatry coordinators for each of the 21 counties in Sweden were contacted with a request to provide e-mail addresses for all directors of outpatient psychiatry clinics in Sweden. For counties where the coordinator did not respond, clinics in the respective counties were identified via a web search and contacted directly. A total of 228 e-mail addresses were collected. In November 2012, an e-mail was sent out to the clinic directors. The e-mail contained information about the purpose of the study and a link to an online survey. The clinic directors were informed about the approximate survey duration (5 min) and were ensured of anonymity. Up to two reminder e-mails were sent if the director did not complete the survey within approximately 2 weeks; reminders were not sent when the director explicitly declined participation. Towards the end of the survey, the clinic directors were asked if they were willing to forward a second online survey to all staff with patient contact at the clinic. Those who indicated willingness to do so were also asked to note the number of staff members they intended to forward the survey to, along with these individuals’ respective professions (e.g., 5 physicians, 3 psychologists, 2 nurses, etc.). Directors willing to forward the survey were then sent an e-mail with a link to the second survey, to be forwarded to all staff members. As for the first survey, the e-mail included information on study purpose, approximate survey duration (10 min), and assurance of participant anonymity. Two reminder e-mails were sent to the directors who had agreed to distribute the e-mail, requesting them to boost staff participation in the study. Given the timing of the survey before the introduction of the DSM-5, DSM-IV terms are used throughout this article.

### Surveys

Both surveys were constructed by the research team and disseminated via SurveyXact, a web survey tool [33]. The clinic director survey consisted of 18 questions covering director and clinic characteristics, clinic guidelines for screening and BI, and possible alternatives for improving clinic practice in relation to identifying and addressing substance use among patients (see Additional file 1). Clinic directors also responded to additional questions regarding practice with these patients. This survey ended with space for optional free text comments on the issue of substance use among patients at the clinic. The staff survey consisted of 38 questions covering staff characteristics, use of screening and BI and possible alternatives for improving clinic practice in relation to identifying and addressing substance use among patients (see Additional file 2). At the end of both surveys, directors and staff were provided three non-mutually exclusive alternatives concerning future improvements: (1) more substance abuse training to staff; (2) improved contact with addiction clinics; and (3) more informational material to patients about substance abuse.

### Statistical analysis

Data from both surveys are presented as simple frequencies. The Chi square test was used to compare directors who agreed to distribute the survey to their staff with directors who did not distribute the survey. We also conducted two secondary analyses on associations between two variables and the use of screening and BI among staff. First, as we considered it reasonable to assume that substance abuse training would be associated with use of screening and BI [34, 35], we used the question regarding experience of substance abuse training (yes/no) as a first variable. Secondly, since prevalence of substance abuse/dependence has been shown to differ markedly depending on psychiatric diagnosis [1], we also used type of clinic as a variable (general/psychosis/specialist). In these secondary analyses, the Chi square test was used. All statistical tests were two-sided with a significance level of 5%, and performed using IBM SPSS Statistics for MacOS X, Version 24 (IBM Corp, Armonk, NY, USA).

## Results

### Respondents

Of the 228 clinic directors who were sent the survey, 57.9% (n = 132) chose to participate. Of these, 46.9% (n = 62) agreed to distribute the staff survey. A comparison between directors who distributed the survey and those who did not showed no significant differences in background characteristics, with the exception of type of clinic; clinic directors at psychosis clinics were more likely to distribute the survey to their staff (77.8%) than clinic directors at specialist clinics (53.1%) and clinic directors at general clinics (43.4%; χ^2^ = 6.966, *df* 2; *p *= 0.031). Of the 1230 staff members estimated to have been forwarded the online link to the staff survey, 42.4% (n = 522) participated. Table 1 shows clinic director and staff characteristics.

**Table 1:** Characteristics of clinic directors and staff who responded to the surveys

Characteristics		Clinic directors (n = 132)		Staff (n = 522)	
Age	Mean (SD)	54.0 (8.2)		48.5 (10.8)	
		%	n	%	n
Gender	Male	22.7	30/132	22.8	119/520
Type of clinic	General	58.3	77/130	56.7	295/520
	Specialist^a^	26.9	35/130	20.5	107/520
	Psychosis	13.8	18/130	22.6	118/520
Substance abuse training^b^	Yes	79.2	103/130	35.7	183/512

^a^In this category we included all specialist clinics other than psychosis (bipolar disorder/obsessive compulsive disorder (OCD)/neuropsychiatric clinics etc.)

^b^‘Yes’ in the clinic director survey indicated any continuing education (CE) training; ‘Yes’ in the staff survey indicated any CE training beyond undergraduate studies

Staff were asked to provide information on their clinical profession and length of experience in psychiatry; 32.1% (n = 166/517) were nurses, 20.3% (n = 105/517) were psychologists, 17.4% (n = 90/517) were mental health workers, 9.7% (n = 50/517) were psychiatrists, 7.2% (n = 37/517) were occupational therapists and 2.1% (n = 11/517) were physiotherapists. Concerning experience, 61.7% (n = 319/517) had more than 10 years of experience working in psychiatry, 16.4% (n = 85/517) had between 6 and 10 years’ experience and 21.9% (n = 113/517) had less than 5 years’ experience.

### Use of screening and brief intervention

#### Clinic directors

Among the clinic directors, 93.1% (n = 121/130) reported having guidelines for clinicians to screen for alcohol use during the initial assessment phase; 78.9% (n = 101/128) reported having such guidelines for illicit substance use (see Table 2). Further, 50.0% (n = 64) reported having guidelines stipulating that staff should provide BI when identifying hazardous alcohol use, while guidelines for providing BI when identifying patients with alcohol abuse/dependence were reported by 35.9% (n = 46). The corresponding figures for illicit substance use were 41.7% (n = 53) for hazardous use and 31.0% (n = 39) for abuse/dependence.

**Table 2:** Screening and brief intervention for alcohol and illicit substance use among clinic directors and staff

Clinic directors’ reports			Staff reports		
Alcohol	%	n	Alcohol	%	n
Having guidelines for alcohol assessment	93.1	121/130	Assessing alcohol use	66.6	259/389
Reported having guidelines for use of BI for hazardous alcohol use	50.0	64/128	Reported use of BI for hazardous alcohol use	36.7	177/482
Reported having guidelines for use of BI for alcohol abuse/dependence	35.9	46/128	Reported use of BI for alcohol abuse/dependence	33.8	162/480
Illicit substances	%	n	Illicit substances	%	n
Reported having guidelines for alcohol assessment	78.9	101/128	Reported illicit substance use assessment	57.8	199/344
Reported having guidelines for use of BI for hazardous illicit substance use	41.7	53/127	Reported use of BI for hazardous illicit substance use	35.7	158/443
Reported having guidelines for use of BI for illicit substance abuse/dependence	31.0	39/126	Reported use of BI for illicit substance abuse/dependence	32.7	145/443

#### Staff

Among staff members, 76.4% (n = 391/512) performed clinical assessment as part of their everyday routines at the clinic; 66.6% (n = 259) of these reported routinely screening for alcohol use during assessment and the corresponding figure for illicit substance use was 57.8% (n = 199). A questionnaire or structured interview was used by 67.9% (n = 258/380) when screening for alcohol use, and by 56.4% (n = 190/337) when screening for illicit substance use. The questionnaire most frequently used when screening for alcohol use was the AUDIT (95.3%; n = 245) [14, 36] and for illicit substance use it was the DUDIT (94.2%; n = 179) [26]. Furthermore, 36.7% (n = 177) reported that they used BI when hazardous alcohol use was identified and 33.8% (n = 162) reported using BI when abuse/dependence was identified; corresponding figures for illicit substance use were 35.7% (n = 158) for hazardous use and 32.7% (n = 145) for abuse/dependence.

### Secondary analyses

#### Associations between substance abuse training and screening/brief intervention

Staff members who reported having received professional substance abuse training were significantly more likely to screen for alcohol use, but substance abuse training was not associated with increased likelihood of screening for illicit substance use (see Table 3). Nonetheless, staff members with substance abuse training were significantly more likely to provide BI following identification of patients with hazardous and abuse/dependence of both alcohol and illicit substances.

**Table 3:** Association between substance abuse training and use of screening and brief intervention among staff

	Staff with substance abuse training				*χ*^*2*^ (df 1)	*p*
	Yes		No			
	%	n	%	n		
Alcohol						
Reported alcohol assessment	75.2	115	61.0	144	8.348	0.004
Reported use of BI for hazardous alcohol use	44.8	77	32.3	100	7.450	0.006
Reported use of BI for alcohol abuse/dependence	43.9	75	28.2	87	12.142	< 0.001
Illicit substances						
Reported illicit substance use assessment	64.0	89	53.7	110	3.653	0.056
Reported use of BI for hazardous illicit substance use	43.3	71	31.2	87	6.601	0.010
Reported use of BI for illicit substance abuse/dependence	40.2	66	28.3	79	6.675	0.010

#### Associations between clinic type and screening/brief intervention

Staff members at psychosis clinics were significantly less likely to screen for both alcohol use and illicit substance use (40.3% and 32.8% respectively) compared to staff at general (73.4% vs 62.0%) and specialist clinics (72.2% vs 68.0%). However, use of BI did not significantly differ between types of clinics. See Table 4.

**Table 4:** Association between type of clinic and use of screening and brief intervention among staff

	Type of clinic						*χ*^*2*^ (*df 2*)	*p*
	General		Specialist		Psychosis			
	%	N*	%	N*	%	N*		
Alcohol								
Reported alcohol assessment	73.4	171_a_	72.2	57_a_	40.3	31_b_	29.933	< 0.001
Reported use of BI for hazardous alcohol use	38.7	106	35.4	35	33.0	36	1.175	0.556
Reporting use of BI for alcohol abuse/dependence	33.9	93	34.7	34	32.4	35	0.131	0.937
Illicit substances								
Reported illicit substance use assessment	62.0	127_a_	68.0	51_a_	32.8	21_b_	21.036	< 0.001
Reported use of BI for hazardous substance use	37.1	93	34.7	33	33.0	32	0.549	0.760
Reported use of BI for illicit substance abuse/dependence	33.5	84	30.5	29	33.0	32	0.274	0.872

* The subscripts a and b indicate which groups significantly differed from one another

### Suggested improvements for clinical practice

Among clinic directors, 69.0% (n = 87/126) recommended provision of substance abuse training to staff, 49.2% (n = 62/126) recommended improved contact with addiction centers and 41.3% (n = 52/126) recommended providing patients with informational material about substance abuse/dependence. Further, 48.4% (n = 61/126) stated that the clinic presently had specially designated staff with specific knowledge about alcohol and illicit substances, and 70.6% (n = 89/126) stated that the clinic currently had a local agreement regarding collaboration with a specialized addiction clinic. Among staff members, 82.5% (n = 359/435) recommended provision of substance abuse training to staff, 60.9% (n = 265/435) recommended improved contact with addiction centers and 47.1% (n = 205/435) recommended providing patients with informational material about substance abuse.

## Discussion

The main purpose of this study was to assess the existence of guidelines for screening and BI for substance use (alcohol and illicit substances) at psychiatric outpatient clinics in Sweden, as well as actual use of screening and BI among staff at these clinics. Almost all clinic directors who responded to the survey indicated that they had clear guidelines for staff to screen for alcohol use and illicit substance use during initial assessment, suggesting high level of awareness in psychiatric clinics about the importance of identifying substance abuse and dependence. However, only about two-thirds of staff members stated that they routinely screened for alcohol during initial assessment, and even fewer stated that they routinely screened for illicit substances. Furthermore, about half of responding clinic directors indicated that they had guidelines for offering BI, while about one-third of staff members indicated that they actually used BI regularly, suggesting that BI is not an integrated part of psychiatric treatment in Sweden.

There are several limitations to this study, most of them related to selection bias. First, with an attrition rate of about 40% among the clinic directors approached, those responding to the survey may have differed in some unknown way from those who did not respond. For example, those clinic directors who chose to respond to the survey may have had more experience of, or knowledge about, substance use and its relevance in a psychiatric context. Those who chose not to respond may have been those who knew that their clinic lacked guidelines. Secondly, only about half of clinic directors responding were willing to distribute the survey to their staff, a factor that may have introduced selection bias in the subsequent staff survey. Directors at psychosis clinics were more willing to distribute the survey, but no other differences were identified when comparing directors who agreed to distribute the survey to their staff, with those who did not. Accordingly, these two groups could have differed in some other way that was not measured. For example, those who chose to distribute the survey may have had more knowledge or experience of working with substance use issues. Also, the clinic directors who chose to distribute the survey could hypothetically have selectively distributed the survey only to a certain subsection of their staff, for example to those at the clinic whom they knew worked with or were more familiar with substance use. Thirdly, almost 60% of staff members who were estimated to have been sent the survey did not respond. We do not know to what extent staff members who responded to the survey were representative of the staff population at large. It could be that staff members who considered substance use an important issue were the ones who took the time to answer the survey, they may have had more experience in working with these issues, or they may have had more substance abuse training. A final limitation relates to the anonymous nature of the staff survey; since we did not ask staff members to indicate where they worked, we have no information about which specific clinics they were working at. There may have been some clinics where no staff responded and other clinics where all or most staff members responded.

Constructive management of patients with substance use in psychiatric outpatient clinics is a complicated matter, involving decision-making at the clinic level as well as policy decisions in the form of national guidelines over which the individual clinic has no influence. Nevertheless, awareness of a patient’s substance use habits is unquestionably a crucial component of psychiatric treatment, and psychiatric clinics should have a well thought-out plan in place on how to identify and manage patients’ substance use. The clinical research literature consistently suggests that treatment of patients with both a psychiatric disorder and substance abuse/dependence optimally should focus on both disorders, rather than on focusing on just one or the other [37]. Although psychotherapy programs have, with some success, been developed specifically to satisfy this aim [38, 39], integrated treatment is often difficult to offer under real-life conditions given the fact that psychiatry and addiction clinics usually are managed by separate health care entities. Nonetheless, about half of the clinic directors in our survey indicated that they maintained local collaboration with addiction care services.

Use of BI was quite rare in our sample, a finding which perhaps is not surprising. Although BI has been shown to be a safe and effective intervention for alcohol when used in other parts of health care, particularly in primary care [40], its place in psychiatry is still unclear and more trials are needed before any conclusions on efficacy in this population can be drawn. Further, the use of BI for illicit substance use is even less investigated. Thus, research is still lacking as to whether BI is even feasible or safe in a psychiatric population. Further, our finding that increased substance abuse training was the most common recommendation for clinical improvement by both clinic directors and staff suggests that the lack of substance abuse training among staff was perceived as a real problem by both groups. Offering staff substance abuse training could thus potentially be an effective way of increasing adherence to guidelines [41], as relatively brief training sessions have been found to improve staff members’ self-perceived knowledge and attitudes towards working with this issue [34, 42]. Lastly, given the consistently high rates of comorbidity reported among patients with schizophrenia, with up to 50% of patients at some point in their life suffering from substance abuse or dependence [1], and considering the fact that premature mortality among these patients due to medical conditions attributable to substance use is common [43], the apparent low use of screening among staff in psychosis clinics was surprising and seems to suggest a very real problem that needs to be addressed.

## Conclusions

Almost all clinic directors stated that they had clear guidelines for staff to screen for alcohol use and illicit substance use, but use of screening among staff was markedly lower than would be expected. Our results thus suggest a gap between the guidelines that the clinic directors report and actual adherence to guidelines by staff. Substance abuse training among staff is rare and may be a contributing factor to low adherence since those indicating that they had substance abuse training reported performing screening and brief intervention to a greater degree. Provision of substance abuse training to staff in psychiatry might thus be a key factor needed to improve frequency of screening and brief intervention. Clinics specializing in psychosis might need to consider making extra efforts to improve screening practices.

## Additional files

**Additional file 1.** Survey to clinic directors.

**Additional file 2.** Survey to staff.

## Abbreviations

SUD: substance use disorders
AUD: alcohol use disorders
BI: brief intervention
AUDIT: Alcohol Use Disorders Identification Test
DUDIT: Drug Use Disorders Identification Test
NICE: National Institute for Health and Care Excellence
NIAAA: National Institute on Alcohol Abuse and Alcoholism
