# Supplementary material for: Identification and management of alcohol use and illicit substance use in outpatient psychiatric clinics in Sweden: a national survey of clinic directors and staff
Source: Addict Sci Clin Pract. 2019 Mar 6;14:10. doi: 10.1186/s13722-019-0140-x (PMC6404264; doi:10.1186/s13722-019-0140-x)
Supplement: Supplementary file 1 — Additional file 1. Survey to clinic directors. [file 13722_2019_140_MOESM1_ESM.pdf]

Questionnaire designed by Christopher Sundström, Kristina Sinadinovic, Anne H Berman
Karolinska Institutet

Survey to clinic directors of psychiatric outpatient clinics in Sweden – English version

This questionnaire is sent to all clinic directors at psychiatric outpatient clinics throughout Sweden. The aim is to, on a national level, examine how psychiatry identifies and manages patients with problematic use of alcohol and/or illicit substances. The survey is part of a major research project aimed at improving the identification and treatment of these patients. Your answers will be treated confidentially. Results from this survey will be presented at the county level, where responses from individuals or individual clinics will not be identifiable. The survey is estimated to take about 2-5 minutes to respond to.

Thanks in advance!

1. You are
(1) Male
(2) Female

2. How old are you?
____

3. Is your clinic public or private?
(1) Public
(2) Private

4. What county do you work in?
____

5. Are you a clinic director at more than one clinic?
(1) Yes
(2) No

(if ”Yes” on Q5) As you are clinic director at more than one clinic, we would like you to respond to the questions that follow, consistently with one of these clinics in mind.

6. Which of the following alternatives best describe the clinic where you are a clinic director?
(1) General psychiatric clinic
(2) Psychosis Clinic
(3) Neuropsychiatric Clinic
(4) Forensic psychiatric clinic
(5) Other specialist clinic, namely: __________

7. How much professional training in alcohol and/or illicit substance abuse do you have (in addition to undergraduate education)?
(1) A full day or less
(2) 2-3 day days
(3) 4-5 day days
(4) More than 5 consecutive days
(5) I have no education in alcohol and/or illicit substance abuse
(6) Do not know/unsure

8. Do clinicians at your clinic have clear guidelines to always investigate alcohol habits during the initial assessment phase?
(1) Yes, we have clear guidelines to always investigate alcohol habits during assessment
(2) No, we have no guidelines to always investigate alcohol habits during assessment
(3) I am uncertain / I do not know

7. Do clinicians at your clinic have clear guidelines to take any specific actions when a patient has hazardous alcohol use?
(1) Yes, brief intervention (screening and feedback, alcohol diary, identifying risk situations etc)

(2) Yes, other_______
(3) Yes, referral to addiction care, social services or primary care
(4) No, we have no guidelines to take any action
(5) Uncertain / do not know

8. Do clinicians at your clinic have clear guidelines take any specific action when a patient has alcohol abuse or dependence?
(1) Yes, brief intervention (screening and feedback, alcohol diary, identifying risk situations etc)
(2) Yes, other_______
(3) Yes, referral to addiction care, social services or primary care
(4) No, we have no guidelines to take any action
(5) Uncertain / do not know

The following questions are about guidelines on illicit substance use among patients at your clinic. By illicit substance use, we mean both illicit substances and medication (such as sedatives or analgesics) used without prescription or not in accordance with the doctor's recommendations.

9. Do clinicians at your clinic have clear guidelines to always investigate illicit substance use during the initial assessment phase?
(1) Yes, we have clear guidelines to always investigate illicit substance use during assessment
(2) No, we have no guidelines to investigate illicit substance use during assessment
(3) Uncertain / Do not know

10. Do clinicians at your clinic have clear guidelines take any specific action when a patient has a hazardous illicit substance use?
(1) Yes, brief intervention (screening and feedback, alcohol diary, identifying risk situations etc)
(2) Yes, other_______
(3) Yes, referral to addiction care, social services or primary care
(4) No, we have no guidelines to take any action
(5) Uncertain / do not know

11. Do clinicians at your clinic have clear guidelines to take any specific action when a patient has illicit substance abuse or dependence?
(1) Yes, brief intervention (screening and feedback, alcohol diary, identifying risk situations etc)
(2) Yes, other_______
(3) Yes, referral to addiction care, social services or primary care
(4) No, we have no guidelines to take any action
(5) Uncertain / do not know

12. Does your clinic have specially appointed staff with knowledge about problematic alcohol and/or illicit substance use?
(1) Yes
(2) No
(3) Uncertain / do not know

13. Does your clinic have a local agreement with a nearby addiction clinic/social services?
(1) Yes
(2) No
(3) Uncertain / Do not know

14. When it comes to identification and management of patients with alcohol and/or illicit substance problems in psychiatry, what do you think could be improved in the work of your clinic? You can note more than one alternative.
(1) Better contact with addiction care
(2) More education to healthcare professionals about alcohol and illicit substances
(3) More information for patients
(4) Other: __________

(5) Nothing, everything works optimally

15. Would you consider forwarding a survey about identification and management of patients with a problematic alcohol or illicit substance use, to clinicians at your clinic? The survey is estimated to take about 5-8 minutes to complete.

(1) Yes, send me the link!
(2) No, thank you

Thanks! We will shortly send you a link to the questionnaire that you can forward to healthcare professionals at your clinic. In order to complete the statistical analysis, we would like you to specify how many individuals in each profession (regardless of scope of service) available at your clinic and whom you will forward the survey to. If you are the clinic director at multiple sites, we are grateful if you enter the number of clinics you had in mind when completing the survey. We are aware that this may take a few minutes to fill in, and are extremely grateful for your help! Note that the results of the survey will only be analyzed at the county level. Responses from individual individuals or clinics will not be identified.

If you are a clinic director at more than one clinic, we would appreciate it if you would name the number of the staff members at the clinic that you have had in mind when responding to the survey.

Medical doctors: ___
Psychologists: ___
Social workers: ___
Nurses: ___
Physiotherapists: ___
Occupational therapists: ___
Mental health worker: ___
Other education: ___

16. Our research team will in the coming years develop e-health services aimed at patients in psychiatry with alcohol and/or illicit substance problems. Would your clinic be interested in collaborating with us? If interested, we will contact you during the spring of 2013 with more information.

(1) Yes, contact me!
(2) No thank you
