# Supplementary material for: Identification and management of alcohol use and illicit substance use in outpatient psychiatric clinics in Sweden: a national survey of clinic directors and staff
Source: Addict Sci Clin Pract. 2019 Mar 6;14:10. doi: 10.1186/s13722-019-0140-x (PMC6404264; doi:10.1186/s13722-019-0140-x)
Supplement: Supplementary file 2 — Additional file 2. Survey to staff. [file 13722_2019_140_MOESM2_ESM.pdf]

Questionnaire designed by Christopher Sundström, Kristina Sinadinovic, Anne H Berman
Karolinska Institutet

Survey to staff members in psychiatric outpatient clinics in Sweden – English version

This questionnaire is sent to healthcare professionals at psychiatric outpatient clinics throughout Sweden. The aim is to, on a national level, examine how psychiatry identifies and manages patients with problematic use of alcohol and/or illicit substances. The survey is part of a major research project aimed at improving the identification and treatment of these patients. Your answers will be treated confidentially and presented at the county level. Replies from individuals or individual clinics will not be identifiable. The survey is estimated to take about 5-8 minutes to respond. Your participation is of course optional, and you can cancel the survey at any time. However, before you begin responding to the survey, we want to inform you that your answers are not saved until the end of the survey. If you close this window before completing the survey, the answers you have left will be deleted. We therefore appreciate if you have the opportunity to complete the survey at one and the same time!

Thanks in advance!

1. You are:
(1) Man
(2) Woman

2. How old are you?
____

3. Is your clinic public or private?
(1) Public
(2) Private

4. Which county do you work in?
____

3. Which of the following alternatives best describes the clinic where you work?
(1) General psychiatric clinic
(2) Neuropsychiatric clinic
(3) Psychosis clinic
(4) Forensic psychiatric clinic
(5) Acute/inpatient clinic
(6) Other specialist clinic, namely: __________

4. What is your profession?
(1) Medical doctor
(2) Psychologist
(3) Social worker
(4) Nurse
(5) Physiotherapist
(6) Occupational Therapist
(7) Mental health worker
(8) Other: __________

5. How many years of work experience do you have in psychiatry?
(1) Less than one year
(2) 1-5 years
(3) 6-10 years
(4) 11-20 years
(5) 21-30 years
(6) 31 years

6. How many individual patient visits do you have a typical working week? Consider the last year. If you have a part-time employment, respond to how many individual visits you would have if you worked full time.
(1) 1-10
(2) 11-20
(3) 21-30
(4) 31-40
(5) 41-
(6) I do not see patients

7. Do you have any professional training in alcohol and/or illicit substance abuse (in addition to undergraduate education)?
(1) Yes
(2) No
(3) Uncertain / Do not know

8. (if "Yes" on Q7) How much professional training in alcohol and/or illicit substance abuse have you received within the framework of your employment in psychiatry over the last five years? Enter total amount in days.
(1) A full day or less
(2) 2-3 full days
(3) 4-5 full days
(4) More than 5 consecutive days
(5) Have not received any professional training within the framework of my employment in psychiatry
(6) Do not know / unsure

9. In psychiatric clinics, assessment interviews are usually held before decisions about treatment are taken. Do you conduct such assessment interviews with patients?
(1) Yes
(2) No

If "Yes" on Q9, continue to Q10
If "No" on Q9, skip to Q13

Questions about alcohol use
In this section of the survey, we would like you to answer questions about problematic alcohol use among patients at your clinic.

10. How often do you investigate alcohol habits during the initial assessment phase?
(1) I always investigate alcohol habits during assessment
(2) I sometimes investigate alcohol habits, depending on whether I suspect there are problematic alcohol habits
(3) I never investigate alcohol habits during assessment
(4) Uncertain / Do not know

11. When you investigate alcohol habits during the initial assessment phase, do you usually use some form of questionnaire/structured interview?
(1) Yes, I use a questionnaire/structured interview
(2) No, I don't use a questionnaire/structured interview

12. (if yes on Q11) Which questionnaire / structured interview do you use? (If several, enter all)
(1) AUDIT
(2) AUDIT-C
(3) CAGE
(4) MAST
(5) ASSIST
(6) Alcohol-E
(7) MINI interview

(8) SCID
(9) SCAN
(10) Other, which? __________

13. What actions do you usually take if you notice that a patient has a hazardous alcohol use? By hazardous use, we mean alcohol use at a level that carries an increased risk of harmful physical, mental and social consequences, but which cannot be said to be abuse or dependence. You can enter several options.
(1) Provide brief intervention (eg screening and feedback, alcohol diary, identification of risk situations, etc.)
(2) Provide information on the negative effects of alcohol
(3) Try to open a dialogue with the patient about alcohol habits
(4) Offer contact with other staff at clinic
(5) Offer referral to other healthcare providers, eg addiction care
(6) I do not take any action
(7) Other, what: ____

14. What actions do you usually take if you notice that a patient has an alcohol abuse or dependence? By abuse or dependence we mean a use of alcohol that causes harmful consequences and recurring problems in relation to the environment. You can enter several options.
(1) Provide brief intervention (eg screening and feedback, alcohol diary, identification of risk situations, etc.)
(2) Provide information on the negative effects of alcohol
(3) Try to open a dialogue with the patient about alcohol habits
(4) Offer contact with other staff at clinic
(5) Offer referral to other healthcare providers, eg addiction care
(6) I do not take any action
(7) Other, what: ____

15. Of the patients you have an established treatment contact with, how many in percent (%) would you estimate have hazardous alcohol use?
Percentage of patients who have a hazardous alcohol use__________

16. Of the patients you have an established treatment contact with, how many in percent (%) would you estimate have alcohol abuse or dependence?
Percentage of patients who have an alcohol abuse or dependence__________

17. Has it ever happened that you have chosen not to raise the issue of alcohol habits during a treatment even though you thought that the patient might have problematic alcohol use of some kind (hazardous use or abuse/dependence)?
(1) Yes, it has happened
(2) No, it has never happened

18. (If yes on Q17) Why did this happen? You can fill in several options.
(1) I don't have enough time
(2) I do not have enough knowledge about alcohol problems
(3) The patient is not interested in discussing alcohol habits
(4) The patient already has an established contact with other healthcare providers, such as addiction care or social services
(5) Other, state what: ____

19. To what extent do you think you have sufficient knowledge about alcohol and its effects to give your patients adequate support in this area?
(1) To a very large extent
(2) To a fairly large extent
(3) To a fairly low extent
(4) To a very low extent

20. How comfortable do you feel about asking your patients about their alcohol habits if necessary?
(1) Very comfortable
(2) Pretty comfortable
(3) Pretty uncomfortable
(4) Very uncomfortable

Questions about illicit substance use
In this section of the survey, we would like you to answer questions about problematic illicit substance use among patients at your reception. With illicit substance use, we mean both illicit substances and medicine (such as sedatives or analgesics) used without prescription or not in accordance with the doctor's recommendations.

If "No" on Q9, skip to Q24.

21. How often do you investigate illicit substance use during the initial assessment phase?
(1) I always investigate illicit substance use during assessment
(2) I investigate illicit substance use sometimes, depending on whether I suspect there might be illicit substance use
(3) I never investigate illicit substance use during assessment
(4) Uncertain / Do not know

22. When you investigate illicit substance use during the initial assessment phase, do you usually use a questionnaire / structured interview?
(1) Yes, I use a questionnaire / structured interview
(2) No, I do not use questionnaire / structured interview

23. (if "Yes" on Q22) Which questionnaire / structured interview do you use? (If several, enter all)
(1) DUDIT
(2) DUDIT-E
(3) MINI International Neuropsychiatric Interview
(4) SCID
(5) SCAN
(6) Other, which? ____

24. What actions do you usually take if you notice that a patient has hazardous illicit substance use? By hazardous use, we mean use that carries an increased risk of harmful physical, mental and social consequences, but cannot be said to be abuse or dependence. You can fill in several options.
(1) Provide brief intervention (eg screening and feedback, illicit substance diary, identification of risk situations, etc.)
(2) Provide information on negative effects of illicit substance use
(3) Try to initiate a dialogue with the patient on illicit substance use
(4) Offer contact with other staff on reception
(5) Offer referral to other healthcare providers, eg addiction care or social services
(6) I do not take any action
(7) Other, what: ____

25. What actions do you usually take if you notice that a patient has illicit substance abuse or dependence? By illicit substance abuse or dependence we mean an intensive use of illicit substances that causes harmful consequences and recurring problems in relation to the environment. You can fill in several options.
(1) Provide brief intervention (screening and feedback, illicit substance diary, identification of risk situations etc.)
(2) Provide information on negative effects of illicit substance use
(3) Try to initiate a dialogue with the patient on illicit substances / illicit substance habits.
(4) Offer contact with other staff at the reception.
(5) Offer referral to other healthcare providers, eg addiction care or social services
(6) I do not take any action
(7) Other, what: ____

26. Of the patients you have an established treatment contact with, how many in percent (%) would you estimate have hazardous illicit substance use?
Percentage of patients who have a hazardous illicit substance use__________

27. Of the patients you have an established treatment contact with, how many in percent (%) would you estimate have illicit substance abuse or dependence?
Percentage of patients who have a illicit substance abuse or dependence___________

28. Has it ever happened that you have chosen not to address illicit substance use even though you thought that the patient might have a problematic illicit substance use that is either hazardous, abuse or dependence)?
(1) Yes, it has happened
(2) No, it has never happened.

29. Why did it happen? You can fill in several options.
(1) I don't have the time
(2) As a clinician, I do not have enough knowledge about illicit substance use
(3) The patient is not interested in discussing illicit substance use
(4) The patient already has an established contact with addiction care/social services
(5) Other, what: ____

30. To what extent do you think you have adequate knowledge about illicit substances and their effects to be able to give your patients adequate support in this area?
(1) To a very high degree
(2) To a quite high degree
(3) To a very low extent
(4) To a very low extent

31. How comfortable do you feel about asking your patients questions about their possible illicit substance use?
(1) Very comfortable
(2) Pretty comfortable
(3) Quite uncomfortable
(4) Very uncomfortable

32. How important do you think it is, in view of your profession and role, to identify problematic use of alcohol / illicit substances among your patients ?
(1) Very important
(2) Pretty important
(3) Pretty unimportant
(4) Very unimportant
(5) Do not know / Unsure

33. Overall how do you think identifying and managing problematic use of alcohol and illicit substances among patients at your clinic is working presently?
(1) Very well
(2) Fairly well
(3) Quite poorly
(4) Very poorly
(5) Do not know / Unsure

34. When it comes to identification and management of patients with alcohol and illicit substance use problems in psychiatry, what do you think could be improved in the work at your clinic? You can enter several options.
(1) More training to staff on mental and somatic consequences of alcohol and illicit substances
(2) More training for staff on therapy methods in substance abuse.

(3) Improved contact with addiction or social services.
(4) Written information material to offer patients
(5) Clearer management decisions on how to deal with problematic use of alcohol / illicit substances
(6) Nothing, the clinic works satisfactorily in this area
(7) Other, what: ____

35.I f you have additional views on problematic Use of alcohol/illicit substances among patients in psychiatry we are grateful if you share them below
.____
____
_____________________________________ ____

36. Our research team at Karolinska Institutet will, in the near future, develop screening and treatment via the Internet, mobile apps, etc. for patients with alcohol and illicit substance problems in psychiatry. We are now seeking psychiatric staff members who would like to participate in interviews or focus groups on this. Would you be interested in attending?
(1) Yes, you can contact me.
(2) No, thank you.

Thank you for your interest! We will contact you in the spring of 2013. In order for us to contact you in our spring, we need your contact details.
Please enter: Email address: _____________________
Confirm your email address: _____________________

The survey is now over. Thanks for taking the time to participate!
